# Use of Skeletal External Fixation to Improve Cultured Epidermal Autograft Outcomes in Lower Extremity Burns

**DOI:** 10.3390/ebj7030043

**Published:** 2026-08-13

**Authors:** Sofie Hass, Cole Bird, Dhaval Bhavsar

**Affiliations:** Department of Plastic Surgery, University of Kansas Medical Center, Kansas City, KS 66160, USA; cbird4@kumc.edu

**Keywords:** cultured epidermal autograft, external fixation, burn, case report

## Abstract

**Highlights:**

**What are the main findings?**

- Skeletal external fixation was successfully used to suspend the lower extremities, minimizing shear and pressure on cultured epidermal autografts (CEA) in two patients with burns involving more than 80% total body surface area.
- Both patients achieved near-complete CEA take, durable epithelial coverage, and excellent functional recovery without external fixation-related complications.

**What are the implications of the main findings?**

- Skeletal external fixation may serve as a simple and effective adjunct to improve CEA adherence and wound healing in extensive lower extremity burns.
- This technique may expand reconstructive options for patients with limited donor site availability by improving the reliability of cultured epidermal autograft treatment.

**Abstract:**

Background: Large surface area burn injuries compromise skin integrity and are associated with significant morbidity and mortality. Skin autografts are essential for wound healing but are limited in patients with extensive burns due to insufficient donor site availability, resulting in delays in wound coverage. Cultured epidermal autograft (CEA) provides burn wound coverage in patients with extremely limited donor sites, but its success is limited by fragility, lack of basement membrane–anchoring cells, and susceptibility to shear and pressure, particularly over the lower extremities. Methods: We present two adult patients with total body surface area burns greater than 80% who underwent treatment with CEA and external fixation of the lower extremities. Burn wounds were excised and temporarily covered with allograft skin prior to CEA application. A triplanar external fixation device was used to suspend the lower extremities off the bed surface to minimize shear and pressure on CEA-treated areas. Results: Following application, both patients demonstrated near-perfect graft adherence, with wounds healing within four weeks and durable epithelial coverage at 12 weeks. No complications related to external fixation were observed. The use of external fixation to suspend the lower extremities reduced shear forces and improved CEA take. This approach provided stable wound healing, facilitated wound care, and demonstrated excellent functional recovery. Conclusions: External fixation may serve as a valuable adjunct in improving outcomes of CEA in patients with extensive burn injuries involving the lower extremities.

## 1. Introduction

The skin is the body’s largest organ and a critical barrier against pathogens. Large surface area burn injury highlights the consequences of compromised skin integrity, leading to significant morbidity and mortality [1]. Skin autografts, essential for achieving wound healing, are limited in availability in patients with large-surface-area burn injuries [2]. Due to the lack of available skin graft donor sites, patients have to wait for previously procured donor sites to heal. This leads to a significant delay in the coverage of these wounds [2]. In patients with extremely large surface-area burn injuries, there are not adequate donor sites available to achieve wound coverage in a timely fashion, even with repeated harvests from the same donor areas [3]. With advances in tissue engineering, cultured epidermal autografts (CEA) were developed in the mid-1970s and entered clinical use for the treatment of extensive burn injuries in the early 1980s [4].

CEA is a technique in which patients’ own epidermal cells are cultured to produce cohesive epidermal sheets [3]. CEA is used to provide burn wound coverage for patients with large surface-area burn injuries when extremely limited skin-graft donor-site availability limits the ability to achieve faster wound healing [3]. CEA adheres to the wound differently than traditional skin grafts. Unlike split-thickness skin grafts, CEA lacks a dermal component and depends on gradual adherence and epithelial maturation rather than the revascularization process characteristic of conventional skin grafts [5]. Even though CEA is fragile and requires very careful handling compared to traditional split-thickness skin autografts, it plays a pivotal role in the care of patients with catastrophically large surface-area burns [3]. In our clinical experience, CEA applied to dependent areas of the lower extremities may be more susceptible to shear-related disruption because of increased mechanical stress and pressure during the early postoperative period.

CEA promotes fast wound healing, reducing scar formation and minimizing infection risk [3]. One major limitation of the CEA graft success rate is the lack of basement membrane-anchoring cells, which hinders stability [5]. Careful wound care strategies are used after CEA application to minimize shear forces. Care is taken to avoid direct pressure over the CEA-grafted areas. Unfortunately, even with the best effort, it is impossible to avoid pressure and shear over dependent areas. External skeletal fixation (EF) involves a rigid frame that stabilizes and supports extremities in the desired position. We used the EF system to suspend the lower extremities off the mattress and support them, reducing the risk of shear and pressure on the CEA-treated areas. In this case study, we present our experience treating two adult patients with TBSA >85% who underwent CEA and EF of the lower extremities, with excellent CEA take. The objective of this case report is to describe the use of skeletal external fixation as an adjunct to cultured epidermal autograft treatment in two patients with extensive burns, highlighting its potential role in achieving complete immobilization, minimizing pressure and shear, and facilitating postoperative wound care.

### 1.1. Case 1

A 59-year-old male suffered 83% TBSA burn injury from a leak of high-pressure steam at work. Burn injury involved both lower extremities, both upper extremities, and most of the anterior and posterior trunk. Areas over the trunk were mostly deep partial-thickness burns, with some full-thickness burns. Wounds over both upper and lower extremities were full-thickness injuries. Skin graft donor sites were available over smaller areas of the upper arm and shoulder, upper back, and left flank. We utilized these donor sites for coverage of burn wounds over bilateral upper extremities, right flank, bilateral groin, posterior upper thighs, and part of the anterior trunk, for full-thickness injuries. Donor sites were used twice to achieve coverage for these areas. Wounds over both lower extremities were excised and initially covered with human allograft skin. We obtained a skin biopsy for growing CEA during our second operating room visit. CEA became available at six weeks post-hospitalization and was subsequently applied at that time based on the patient’s wound-bed readiness and overall clinical readiness for reconstruction.

### 1.2. Case 2

A 30-year-old female suffered 87% TBSA burn injury from a house fire. She had to jump out of the burning building. She also sustained an inhalation injury due to prolonged exposure to smoke within the confined space. Her burn injuries involved most of the trunk and extremities, with the majority being full-thickness. Burns to the face and neck were present, but were not full-thickness. Due to the extensive nature of her injury, we obtained a skin biopsy for CEA very early into her hospitalization. Skin graft donor sites were available on parts of the back, scalp, buttocks, and one shoulder. These donor sites were utilized for coverage of full-thickness burn injuries over both upper extremities, the anterior trunk, parts of the back, and the neck. A small area over the right posterior upper thigh was also covered with widely meshed autograft. Lower extremity burns were excised and covered with allograft skin to temporize these wounds. CEA was available and was planned for application about eight weeks after the burn injury based on her overall clinical readiness for definitive reconstruction and wound-bed readiness for CEA application.

## 2. Methods

### Preparation of Burn Wounds for CEA and Its Application

We followed a similar process in preparing the burn wounds over both lower extremities for CEA application for both patients. One week before the planned application, all burn wounds were excised again and covered with fresh 1:1 meshed allograft skin. Wounds were dressed with gentamicin and polymyxin B solution-soaked gauze over the past week. We planned the timing of the CEA application so that a small amount of skin donor site would be available. The timing of CEA application was individualized based on wound-bed readiness, availability of limited donor skin for widely meshed autografting, and overall clinical readiness for definitive reconstruction rather than CEA culture time alone. A triplanar external fixation device was placed 2 days prior to the planned CEA. The external fixator extended from the thigh to the ankle and suspended the entire lower extremity off the bed surface. The knee was maintained in slight flexion while the ankle was positioned in neutral to minimize soft tissue tension, maintain a functional resting position, and avoid excessive stress on the CEA-treated surfaces throughout the immobilization period. On the day of the CEA application, during surgery, allografts were removed, and burn wounds were examined. All areas were gently debrided again using a pressurized water jet device. This allowed us to ensure that all wounds had healthy vascularity. Available skin graft donor sites were used to obtain a 0.008-inch-thick skin graft. Skin graft was meshed at a 6:1 ratio. Widely meshed autografts were applied to all of the wounds. A thin layer of fibrin glue was sprayed over the wounds. CEA sheets were applied to all areas next. All treated areas were initially covered with nylon mesh, followed by gauze soaked in Gentamicin and polymyxin B solution. An external fixation device allowed the lower extremities to be suspended off the bed surface and prevented pressure or shear on the posterior aspects of the lower extremities (Figure 1). Wound dressings (except nylon mesh) were removed after 48 h, and CEA grafts were exposed to air for 6 h. At the end of six hours, both lower extremities were rewrapped with the same dressing as described above. This was repeated daily with increasing duration of drying (up to 12 h). On day 11 post CEA application, we observed that CEA was adherent. On this day, all the dressings were removed. Nylon mesh and the backing of CEA sheets were gently separated from newly adherent CEA. For both patients, we observed near-perfect CEA take (Figure 2). Graft take was assessed clinically by the surgeon based on the percentage of adherent viable CEA at the time of dressing removal; no standardized quantitative assessment tool was used. Beyond this, the wound was continued to be dressed with a non-adherent layer, followed by gentamicin and polymyxin B solution-soaked gauze for an additional 1 week, with daily drying for at least 10 h (maximum 12 h). After grafts looked secure, we started applying light moisturizer and wrapped them with a non-adherent dressing. The external fixator was removed on day 21 after its application. Gradually progressive mobility was initiated at this time. Scar stability and functional recovery were evaluated clinically by the treating team during follow-up. Scar stability was assessed based on maintenance of stable epithelial coverage without recurrent wound breakdown or need for additional reconstructive procedures, while functional recovery was assessed based on progressive return to ambulation. No standardized scar or functional assessment instruments were used.

## 3. Results

Both patients achieved a near-100% CEA take rate. All involved wounds healed within four weeks. The nursing team did not have to worry about shear forces on the CEA-grafted areas during turns or during care of other burn-injured areas. Lower extremities in a cradled position were easier to care for during the post-CEA application period. The external fixation system also facilitated routine nursing care by minimizing inadvertent shear during patient repositioning and dressing changes while allowing the lower extremities to remain completely off-loaded throughout the early postoperative period. At 12 weeks following CEA application, both patients demonstrated durable epithelial coverage and stable wound healing (Figure 3). Long-term, both patients had excellent scar stability and functional recovery at the six-month follow-up. At six months, both patients demonstrated stable epithelial coverage without recurrent breakdown or need for reconstructive procedures and had returned to progressive ambulation, which formed the basis for our assessment of scar stability and functional recovery. Neither patient required any procedure for scar remodeling or reconstructive procedures for the lower extremities during the late recovery phase. We did not observe any complications during this treatment.

## 4. Discussion

The use of a lower extremity EF significantly contributed to improved CEA take by stabilizing the affected area and minimizing shear forces that could disrupt the attachment of delicate grafts, thereby enhancing the overall success of graft adherence. This finding is biologically plausible given the known fragility of CEA and its reliance on gradual adherence in the absence of a dermal component and mature basement membrane anchoring during early healing [3,5]. Additionally, it allowed better access for wound care, ensuring that dressings could be applied and maintained with minimal disruption of healing.

Our findings are consistent with those reported by Konstantakos et al., who described the use of uniplanar external fixation to protect circumferential lower extremity skin grafts in adult burn patients. Their study demonstrated that external fixation minimized shear forces, facilitated postoperative wound care, and was associated with successful graft healing without significant fixation-related complications. While their experience focused on conventional split-thickness skin grafts, our report extends this concept to cultured epidermal autografts, which are substantially more fragile and susceptible to mechanical disruption during the early postoperative period. These findings suggest that skeletal external fixation may provide an important adjunct for protecting CEA and optimizing graft adherence in patients with extensive burns and limited donor-site availability [6].

Despite the benefits, it is important to acknowledge the inherent risks associated with the use of external fixators, particularly in individuals with extensive open wounds. These risks include potential complications such as infection at the pin sites, stiffness, and discomfort for the patient. Though the incidence of osteomyelitis related to the pin track site is low, it is not insignificant [7]. However, in our experience, the external fixation system appeared feasible in these two patients, with no significant complications reported. This approach demonstrates a promising adjunctive technique that may improve the outcomes of CEA in the management of extensive burn wounds on the lower extremities. A limitation of this report is that formal postoperative knee and ankle range-of-motion measurements were not prospectively collected. Although both patients underwent gradual mobilization following external fixator removal and achieved excellent functional recovery without requiring subsequent reconstructive procedures of the lower extremities, objective joint motion measurements were unavailable for analysis. Future studies should include standardized functional outcome measures, including range-of-motion assessments, to further evaluate the impact of this technique.

Patient selection is an important consideration when using skeletal external fixation as an adjunct to CEA. Based on our experience, this technique may be most beneficial in patients with extensive lower extremity burns, severely limited donor-site availability, and CEA applied to dependent surfaces where complete immobilization and off-loading cannot be reliably achieved using conventional positioning methods alone. In contrast, patients with smaller burn injuries or those in whom adequate pressure relief and shear reduction can be accomplished with standard positioning and splinting strategies may not derive sufficient additional benefit to justify the invasiveness and potential risks associated with external fixation. Therefore, careful patient selection and individualized clinical judgment remain essential when considering this technique.

Our experience underscores the potential of using an external fixation system to enhance the outcomes of CEA in managing extensive burn injuries, particularly for CEA applied to the posterior aspect of the lower extremities, where maintaining complete off-loading and minimizing shear can be especially challenging during postoperative care. The results of this study suggest that a combination of CEA and EF may represent a valuable adjunct in burn care, improving graft success rates and patient recovery. However, successful wound healing in these patients likely reflected the combined effects of meticulous wound-bed preparation, widely meshed autografting, fibrin glue application, structured postoperative wound care, and external fixation. Given the absence of a comparison group, the specific contribution of external fixation to CEA take cannot be determined and warrants further investigation. Future studies evaluating larger patient cohorts with standardized functional and scar outcome measures will help further define the role of skeletal external fixation in patients undergoing cultured epidermal autograft reconstruction.

## 5. Conclusions

This case report suggests that skeletal external fixation may represent a promising adjunct to cultured epidermal autograft application in patients with extensive lower extremity burns. By suspending the lower extremities off the bed surface, external fixation reduced pressure and shear on CEA-treated areas and was associated with near-perfect graft take, durable epithelial coverage, and excellent functional recovery without complications related to the external fixation system. Although this report is limited to two cases, our experience suggests that external fixation may improve CEA outcomes and facilitate postoperative wound care in patients with limited donor site availability. Further studies are warranted to evaluate this technique in a larger patient population.

## Figures and Tables

**Figure 1:**
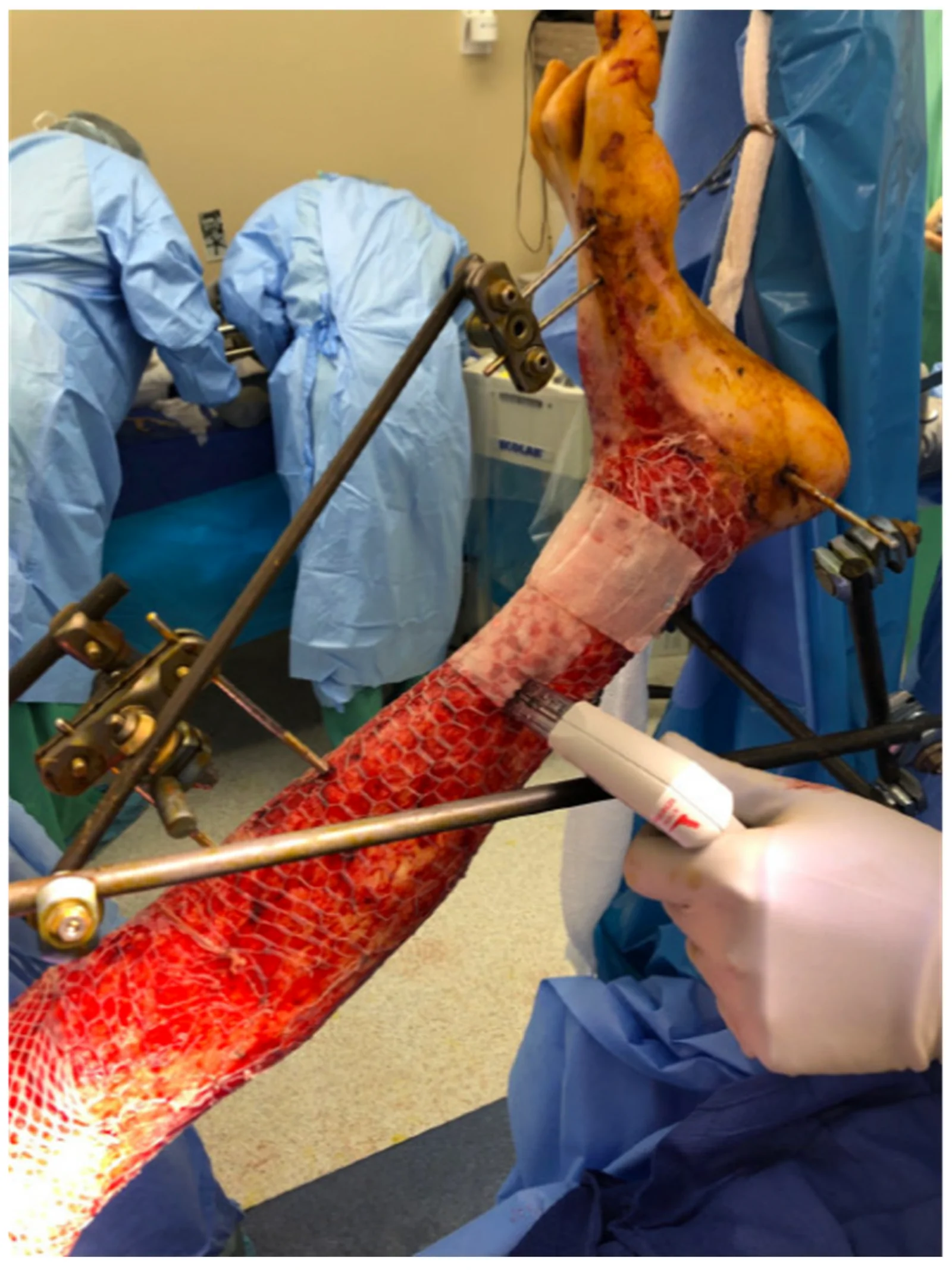
Application of CEA With External Fixation. Intraoperative photograph demonstrating application of CEA to the lower extremity following excision of full-thickness burn wounds. A triplanar external fixation device suspends the limb off the operating surface to minimize pressure and shear during graft placement.

**Figure 2:**
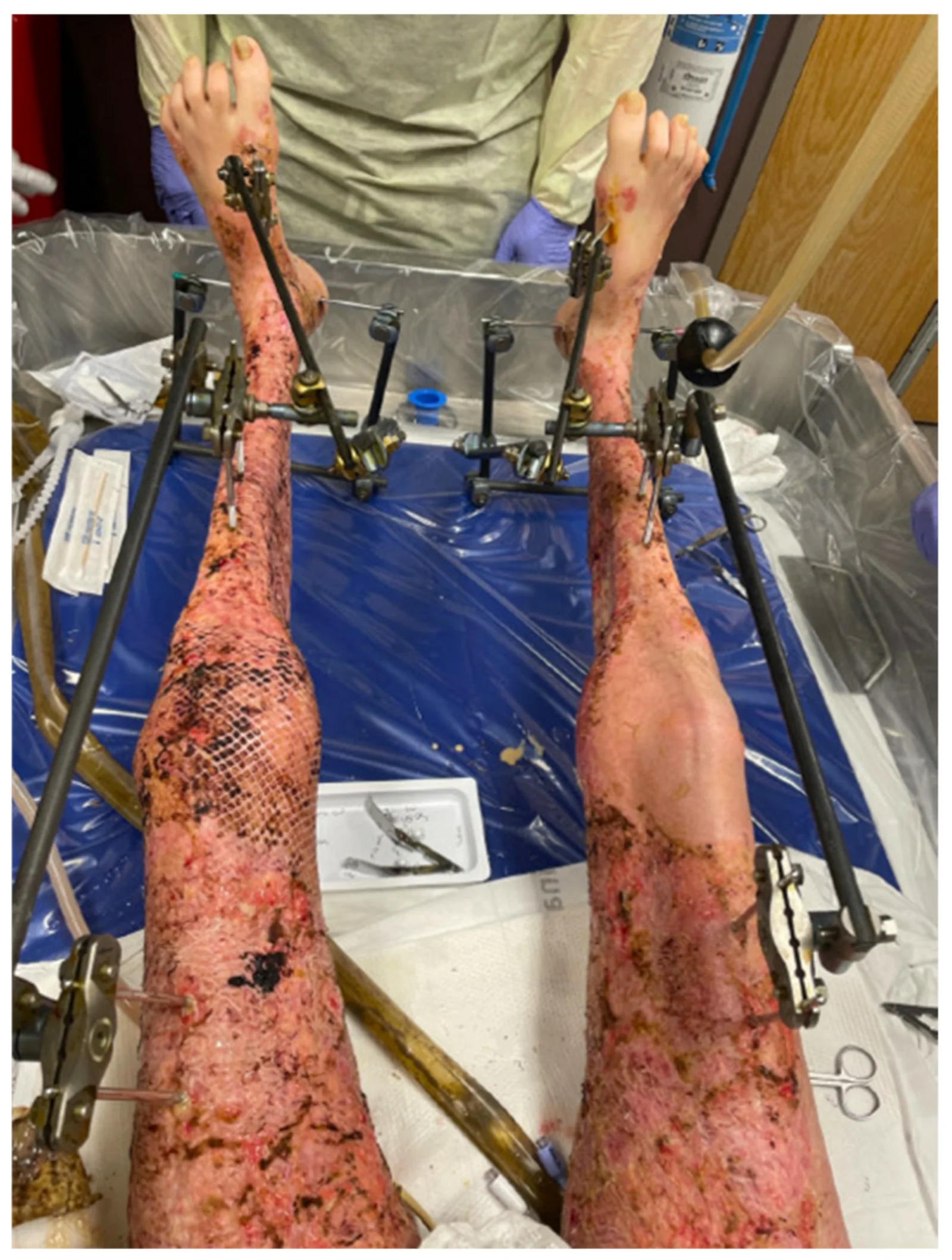
Post-takedown Appearance Following CEA Application. Clinical photograph of bilateral lower extremities on post-operative day 11 following CEA application, demonstrating near-complete graft take and progressive epithelialization with external fixation in place.

**Figure 3:**
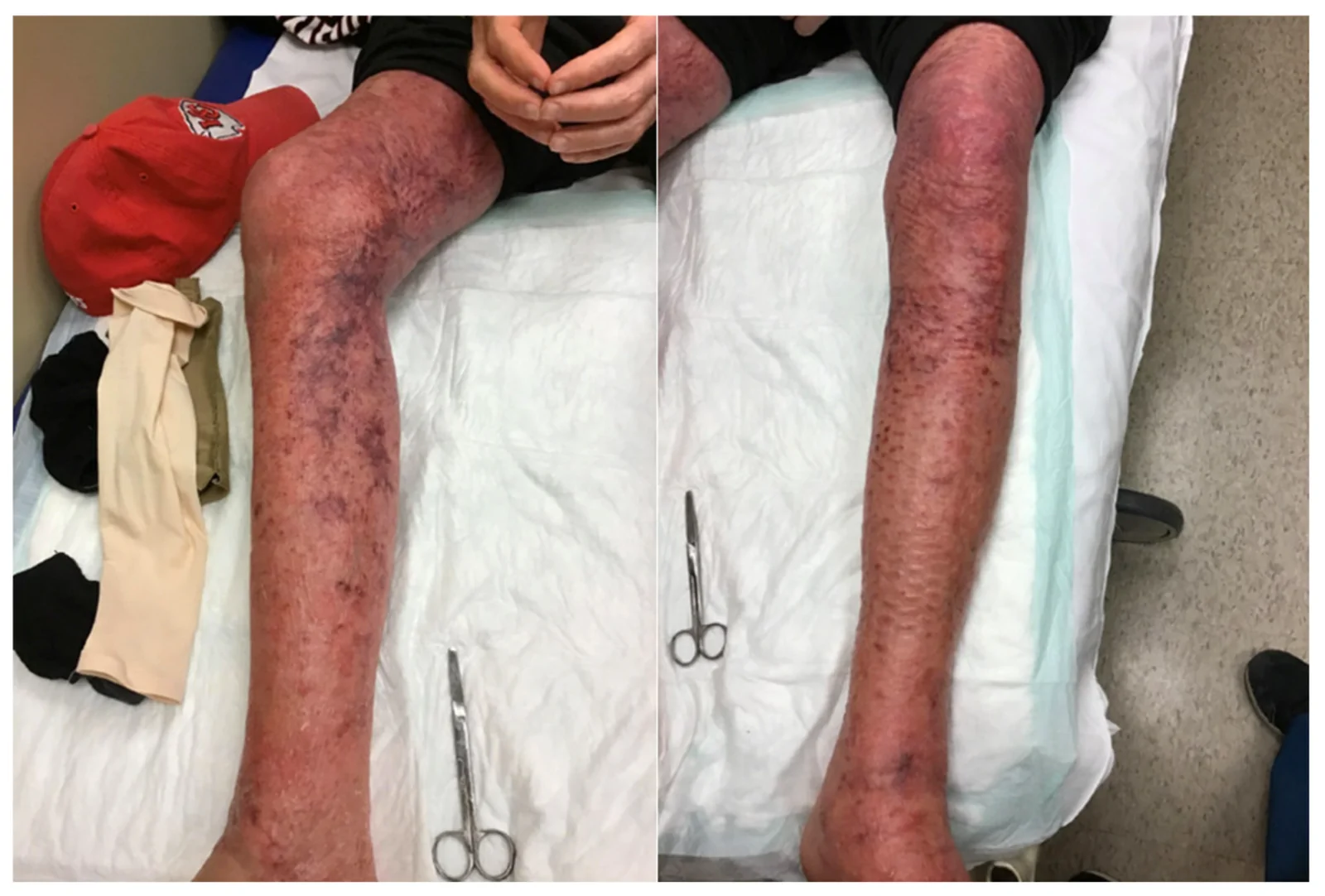
Long-term Outcome Following CEA Application. Clinical photographs of the bilateral lower extremities at 12 weeks following cultured epidermal autograft (CEA) application, demonstrating durable graft take, stable epithelial coverage, and satisfactory long-term wound healing.

## Data Availability

The data presented in this study are available upon request from the corresponding author due to privacy restriction.
